# Population‐Based Prediction Equations for Appendicular Skeletal Muscle Mass: A Three‐Phase Validation Study in 13 582 Asian Adults

**DOI:** 10.1002/jcsm.70360

**Published:** 2026-09-06

**Authors:** Hsi‐Yu Lai, Shu Zhang, I‐Tzu Chen, Rei Otsuka, Wei‐Ju Lee, Chih‐Kuang Liang, Ko‐Han Yen, Hidenori Arai, Fei‐Yuan Hsiao, Liang‐Kung Chen

**Affiliations:** ^1^ Graduate Institute of Clinical Pharmacy, College of Medicine National Taiwan University Taipei Taiwan; ^2^ Center for Healthy Longevity and Aging Sciences National Yang Ming Chiao Tung University Taipei Taiwan; ^3^ National Center for Geriatrics and Gerontology Obu Aichi Japan; ^4^ Department of Family Medicine Taipei Veterans General Hospital Yuanshan Branch Yilan Taiwan; ^5^ Department of Geriatric Medicine National Yang Ming Chiao Tung University School of Medicine Taipei Taiwan; ^6^ Center for Geriatrics and Gerontology Kaohsiung Veterans General Hospital Kaohsiung Taiwan; ^7^ Center for Geriatrics and Gerontology Taipei Veterans General Hospital Taipei Taiwan; ^8^ School of Pharmacy, College of Medicine National Taiwan University Taipei Taiwan; ^9^ Department of Pharmacy National Taiwan University Hospital Taipei Taiwan; ^10^ Taipei Municipal Gan‐Dau Hospital (Managed by Taipei Veterans General Hospital) Taipei Taiwan

**Keywords:** anthropometry, body composition, muscle health, sarcopenia, skeletal muscle mass

## Abstract

**Background:**

Accurate estimation of appendicular skeletal muscle mass (ASM) is crucial for sarcopenia diagnosis, but device‐dependent approaches (dual‐energy X‐ray absorptiometry [DXA] or bioelectrical impedance analysis [BIA]) limit widespread application in community settings. Developing prediction equations using readily available anthropometric measurements and common laboratory data (such as serum creatinine) offers a practical alternative for large‐scale screening. However, no population‐based prediction equations using these accessible parameters have been developed specifically for Asian populations, where body composition differs significantly from Western cohorts.

**Methods:**

We conducted a three‐phase investigation: Phase 1 validated existing NHANES‐derived equations across three cohorts (*n* = 13 582) from Taiwan (ILAS, *n* = 2780; NAHSIT, *n* = 6853) and Japan (NILS‐LSA, *n* = 3949); Phase 2 developed new population‐based equations using the NAHSIT cohort; and Phase 3 performed external validation of newly developed NAHSIT‐derived equations in an independent Japanese cohort (NILS‐LSA). Model performance was assessed using *R*‐squared, root mean square error (RMSE) and area under the receiver operating characteristic curve (AUC) for detecting low muscle mass based on Asian Working Group for Sarcopenia (AWGS) 2019 criteria.

**Results:**

In Phase 1 external validation, existing equations showed acceptable to strong predictive accuracy across populations (*R* ^2^ = 0.52–0.89). Equation 4 (incorporating sex, age, height and weight) demonstrated robust performance across ILAS (*R* ^2^ = 0.80, RMSE = 1.88), NILS‐LSA (*R* ^2^ = 0.86, RMSE = 1.51) and NAHSIT (*R* ^2^ = 0.89, RMSE = 1.52) cohorts. For detecting low muscle mass using AWGS 2019 criteria, AUC values ranged from 0.79 to 0.91 in males and 0.76 to 0.89 in females across cohorts. In Phase 2, our newly developed population‐based Equation 4 achieved superior performance (*R* ^2^ = 0.90, RMSE = 1.45) with minimal prediction bias (median error: 0.06, IQR: 1.81) and excellent diagnostic capability (AUC = 0.90 in both sexes; sensitivity/specificity: 0.85/0.80 in males and 0.87/0.80 in females). Phase 3 external validation in the independent Japanese cohort (NILS‐LSA) demonstrated robust cross‐population generalizability, with Equation 4 achieving the highest AUC values (0.90 in males and 0.85 in females).

**Conclusions:**

Our three‐phase investigation has established and validated the first population‐based prediction equations for ASM estimation in Asian populations. These clinically accessible equations overcome device‐dependency limitations and enable practical, large‐scale sarcopenia screening in community settings, representing a significant advance for early identification of sarcopenia and promotion of healthy muscle aging across Asian populations.

**Abbreviations**
ASM: appendicular skeletal muscle mass
AUC: area under the receiver operating characteristic curve
BIA: bioelectrical impedance analysis
DXA: dual‐energy X‐ray absorptiometry
ILAS: I‐Lan Longitudinal Aging Study
NAHSIT: Nutrition and Health Survey in Taiwan
NHANES: National Health and Nutrition Examination Survey
NILS‐LSA: National Institute for Longevity Sciences‐Longitudinal Study of Aging
RMSE: root mean square error

## 1 Introduction

Sarcopenia, a progressive age‐related skeletal muscle disorder, represents a significant health challenge in community‐dwelling older adults [1, 2, 3].It increases the risk of falls, fractures, hospitalization and mortality in community settings [4, 5] and is associated with reduced vitality and cognitive function [6, 7, 8]. Despite its clinical implications, sarcopenia management remains challenging due to the paucity of efficacious pharmacological agents; consequently, therapeutic approaches mainly rely on lifestyle modifications (exercise and nutrition) to enhance muscle health throughout the aging process [9, 10, 11, 12]. To address this challenge, accurate sarcopenia diagnosis and precise, quantifiable metrics of muscle health are essential for widespread clinical implementation.

Both the European Working Group on Sarcopenia in Older People (EWGSOP) and the Asian Working Group for Sarcopenia (AWGS) [13, 14, 15] have emphasized muscle mass as the core diagnostic element for sarcopenia. Although magnetic resonance imaging (MRI) and computed tomography (CT) provide superior muscle quantification by distinguishing intramuscular fat from contractile tissue and dual‐energy X‐ray absorptiometry (DXA) is routinely used for body composition assessment, these methods have limited community applicability due to accessibility and cost constraints [16]. Bioelectrical impedance analysis (BIA), though portable, faces accuracy challenges due to method‐, device‐, and ethnicity‐dependent variations [16]. Recently, the National Health and Nutrition Examination Survey (NHANES)–based equations for estimating appendicular skeletal muscle mass (ASM) demonstrated excellent performance (*R* ^2^: 0.89–0.91; AUC: 0.90–0.95) in Western populations [17]. However, given the well‐documented ethnic heterogeneity in body composition parameters and anthropometric profiles between Asian and Western populations, the direct extrapolation of these equations to Asian demographic cohorts may be subject to significant validity constraints. Studies from Western countries report significant muscle loss after age 50 in both sexes [18, 19], whereas research from Taiwan, Japan, Korea and Hong Kong indicates that Asian women maintain muscle mass until approximately age 80 [20, 21]. These regional differences in age‐related muscle mass trajectories underscore the need for population‐specific assessment approaches.

To address this knowledge gap, we conducted a comprehensive three‐phase investigation: Phase 1 validated existing NHANES‐derived equations across three cohorts (*n* = 13 582) from Taiwan (ILAS, *n* = 2780; NAHSIT, *n* = 6853) and Japan (NILS‐LSA, *n* = 3949); Phase 2 developed new population‐based equations using the NAHSIT cohort; and Phase 3 performed external validation of the newly developed NAHSIT‐derived equations in an independent Japanese cohort (NILS‐LSA) to demonstrate cross‐population generalizability.

## 2 Methods

### 2.1 Study Design and Overview

We conducted a comprehensive three‐phase investigation to validate existing equations and develop new population‐based prediction models for appendicular skeletal muscle mass (ASM) in Asian populations. Phase 1 externally validated existing NHANES‐derived equations across three Asian cohorts; Phase 2 developed new Asian‐specific equations using the largest cohort; and Phase 3 validated the newly developed equations in an independent population to assess cross‐population generalizability.

### 2.2 Data Source and Study Participants

We extracted data from three cohort studies from Taiwan and Japan: the I‐Lan Longitudinal Aging Study (ILAS) [22], National Institute for Longevity Sciences‐Longitudinal Study of Aging (NILS‐LSA) [23, 24] and Nutrition and Health Survey in Taiwan (NAHSIT) [25].

The ILAS is an ongoing cohort study in I‐Lan County, Taiwan, investigating the interrelationship between aging, sarcopenia, frailty and cognitive decline. Participants aged ≥ 50 years were randomly selected from the community, excluding those with uncontrolled clinical conditions, disability, dementia or terminal illnesses. For ILAS, we included individuals who initially participated in the study at the first wave (*n* = 1839) and third wave (*n* = 1001) to maximize the sample size. We excluded participants with incomplete baseline muscle mass measurement (*n* = 60), resulting in a final analytical cohort of 2780 individuals.

The NILS‐LSA comprises a prospective cohort study examining aging‐related variables in a Japanese population. Based in Aichi Prefecture, the study recruited participants from two municipalities—Obu and Higashiura—using stratified random sampling by age and sex. Beginning between November 1997 and April 2000, the initial survey included 2267 individuals between 40 and 79 years of age. Biennial follow‐up examinations were conducted for all participants. To maintain the age‐sex distribution of the cohort, new participants were randomly selected to replace those under 80 who withdrew from the study. The study design also incorporated annual recruitment of 40‐year‐old participants. For NILS‐LSA, the baseline survey for this analysis comprised participants who initially joined NILS‐LSA during the first seven waves, spanning from November 1997 to July 2012, which encompassed all participant recruitment periods (*n* = 3983). After excluding 34 individuals with incomplete baseline data, the final analytical cohort consisted of 3949 participants.

The NAHSIT is a nationwide, population‐based survey conducted by the Health Promotion Administration (HPA) to monitor the health and nutritional status of Taiwanese citizens [25]. Using a multistage, stratified, clustered probability sampling design, data are collected through in‐person interviews, health measurements and mobile health stations, representing the noninstitutionalized Taiwanese population of all ages. NAHSIT provides critical data on lifestyle behaviours, chronic conditions, nutritional status and public health trends to support evidence‐based policymaking. For the NAHSIT cohort, adult subjects (>18 years) from both survey periods (2013–2020) were included, with subsequent exclusion of those lacking physical examination data or complete baseline covariates, ultimately yielding an analytical sample of 6853 participants.

The study design, recruitment and data collection details of all three cohorts are included in Supporting Information S1.

### 2.3 Measurements and Definitions

- ASM assessment: ASM was assessed using dual‐energy X‐ray absorptiometry (DXA) across all cohorts, defined as the sum of lean soft tissue mass of all four limbs. ILAS and NAHSIT used Lunar Prodigy instruments (GE Healthcare), whereas NILS‐LSA used QDR‐4500 (Hologic).
- Skeletal Muscle Index (SMI): calculated as ASM divided by height squared (kg/m^2^). We also adjusted for the BMI to obtain BMI‐adjusted ASM (ASM/BMI, kg/[kg/m^2^]).
- Low muscle mass definition: based on AWGS 2019 criteria [13, 14] using sex‐specific thresholds: < 7.0 kg/m^2^ for men and < 5.4 kg/m^2^ for women.

### 2.4 Ethical Approval

The Institutional Review Board of National Taiwan University approved the NAHSIT protocol (202307077RIND). The institutional review board of National Yang Ming University (YM103008) and Taipei Veterans General Hospital (2018‐05‐003B) approved the ILAS study protocol. The National Center for Geriatrics and Gerontology Committee on Ethics of Human Research approved the NILS‐LSA protocol (Nos. 1453‐4 and 1665‐3). All participants gave written informed consent before any study‐related procedure ensued.

### 2.5 Phase 1: External Validation of Existing NHANES‐Derived Equations Across Three Diverse Asian Cohorts (ILAS, NILS‐LSA and NAHSIT)

All three cohorts (ILAS, NILS‐LSA and NAHSIT) were utilized for external validation, totalling 13 582 participants. Study participants were deemed eligible for inclusion contingent upon the availability of requisite data elements for ASM estimation according to each predictive equation (age, sex, body height, body mass, serum creatinine concentrations and DXA‐measured ASM measurements) and baseline anthropometric parameters (height and weight).

We selected equations 3–6 from Shi et al. [17] for validation, excluding equations requiring serum cystatin C due to limited clinical accessibility and utility. To maintain consistency and facilitate comparisons with previous studies, we aligned the equation names with prior research [17], as described in Supporting Information S2. The estimated ASM was computed for each existing equation, and model performance was evaluated using *R*‐squared, root mean square error (RMSE), the median and interquartile range of absolute differences between actual and estimated ASM, and the Bland–Altman plot for each equation across the three cohorts.

To evaluate the model's ability to detect low muscle mass, we employed receiver operating characteristic (ROC) curves, measuring its performance using the area under the curve (AUC), sensitivity and specificity.

### 2.6 Phase 2: Development of New Asian‐Specific Prediction Equations From NAHSIT

The NAHSIT cohort (*n* = 6853) served as the foundation for developing new Asian‐specific equations. We split the dataset into development set (70%) and validation set (30%) using random sampling (Figure S1).

The model development was in accordance with the previous methodology [17]. We manually selected candidate variables to be incorporated into the regression model: age, sex, height, weight, serum creatinine, serum urea nitrogen, serum calcium, serum phosphorus, urine protein and urine creatinine [17]. To account for nonlinear relationships between variables, we transformed each variable to a log scale and compared different combinations within a statistical framework in regression analysis. Variables with a *p* value of < 0.01 in the univariate regression in any statistical framework were incorporated into the multivariate regression. Subsequently, only variables with a *p* value of < 0.001 in all statistical frameworks in the multivariate regression were selected for inclusion in the stepwise regression analysis.

We evaluated various statistical equation frameworks to determine the optimal model using the development set by considering *R*‐squared, Akaike information criterion (AIC) and Bayesian information criterion (BIC). After selecting the best equation, we extracted the coefficients of key variables identified through stepwise regression and applied them to the validation set. Model performance was assessed using *R*‐squared, RMSE, the median and interquartile range of absolute differences between actual and predicted ASM, and the Bland–Altman plot. Additionally, we utilized ROC curves to measure performance, focusing on the AUC, sensitivity and specificity.

To improve the generalizability and stability of the model, we incorporated the selected variables obtained from the previous process and extracted coefficients from the full dataset of 6853 participants in NAHSIT. This approach minimizes the potential bias derived from the specific subset of the data and thus provides more robust and reliable estimates to improve the accuracy. We further compared the estimated SMI with the actual SMI by generating the scatter plot and Bland–Altman plot for each equation, which helps us offer a more visual assessment of model performance. These visualizations provide a comprehensive assessment of the agreement between the estimated and actual SMI values, further supporting the diagnostic performance of the proposed equations.

### 2.7 Phase 3: External Validation of Newly Developed Asian‐Specific Equations From NAHSIT in NILS‐LSA

The NILS‐LSA cohort served as an independent external validation dataset to assess the generalizability of our newly developed equations across different Asian populations.

Performance was evaluated using the same metrics as in previous phases: *R*‐squared, RMSE, median and interquartile range of absolute differences, Bland–Altman plot and ROC curve analysis (AUC, sensitivity and specificity) for detecting low muscle mass according to AWGS 2019 criteria. We also assessed the correlation between actual and predicted SMI.

### 2.8 Statistical Analysis

Descriptive analysis of demographic and clinical characteristics was conducted, with continuous variables presented as the mean ± standard deviation (SD) and median with interquartile range (IQR), whereas categorical variables were reported as counts and percentages in both text and tables. Baseline characteristics were compared using the chi‐squared test for categorical variables and the Kruskal–Wallis test for continuous variables when appropriate. All statistical analyses were performed using SAS software (Version 9.4, SAS Institute, NC, United States) and R software (Version 4.4.2). A two‐tailed *p* value of < 0.05 was considered statistically significant.

## 3 Results

### 3.1 Baseline Characteristics Across the Three Cohorts

Table 1 summarizes the baseline characteristics of participants from ILAS (*N* = 2780), NILS‐LSA (*N* = 3949) and NAHSIT (*N* = 6853). We also present sex‐specific characteristics in Table S1 for further comparisons. Across all cohorts, the proportion of female participants was approximately 50%, and mean height ranged from 159.0 cm (NILS‐LSA) to 161.0 cm (NAHSIT).

**TABLE 1:** Characteristics of the total population across the three cohorts.

Variables	ILAS (*N* = 2780)	NILS‐LSA (*N* = 3949)	NAHSIT (*N* = 6853)
Age (years), mean (SD)	63.5 (8.5)	56.8 (13.0)	56.0 (17.4)
< 25, *n* (%)	0 (0.0)	0 (0.0)	359 (5.2)
25–29, *n* (%)	0 (0.0)	0 (0.0)	413 (6.0)
30–34, *n* (%)	0 (0.0)	0 (0.0)	320 (4.7)
35–39, *n* (%)	0 (0.0)	0 (0.0)	362 (5.3)
40–44, *n* (%)	0 (0.0)	1040 (26.3)	382 (5.6)
45–49, *n* (%)	13 (0.5)	428 (10.8)	433 (6.3)
50–54, *n* (%)	445 (16.0)	380 (9.6)	518 (7.6)
55–59, *n* (%)	571 (20.5)	371 (9.4)	639 (9.3)
60–64, *n* (%)	566 (20.4)	383 (9.7)	653 (9.5)
65–69, *n* (%)	485 (17.4)	319 (8.1)	1155 (16.9)
70–74, *n* (%)	394 (14.2)	615 (15.6)	731 (10.7)
75–79, *n* (%)	197 (7.1)	402 (10.2)	500 (7.3)
80+, *n* (%)	109 (3.9)	11 (0.3)	388 (5.7)
Female, *n* (%)	1425 (51.3)	1982 (50.2)	3454 (50.4)
Height (cm), mean (SD)	159.5 (8.1)	159.0 (9.4)	161.0 (8.9)
Weight (kg), mean (SD)	62.7 (11.0)	57.9 (10.5)	64.2 (12.9)
BMI (kg/m^2^), mean (SD)	24.6 (3.5)	22.8 (3.10)	24.7 (4.1)
≤ 18.4, *n* (%)	44 (1.6)	241 (6.1)	242 (3.5)
18.5–23.9, *n* (%)	1254 (45.1)	2471 (62.6)	2872 (41.9)
24.0–29.9, *n* (%)	1304 (46.9)	1155 (29.2)	2934 (42.8)
30.0–39.9, *n* (%)	176 (6.3)	79 (2.0)	649 (9.5)
≥ 40.0, *n* (%)	2 (0.1)	3 (0.1)	156 (2.3)
ASM (kg), mean (SD)	17.8 (4.2)	17.3 (4.1)	18.1 (4.6)
SMI (kg/m^2^), mean (SD)	6.9 (1.1)	6.7 (1.0)	6.9 (1.2)
ASM/BMI, kg/(kg/m^2^), mean (SD)	0.7 (0.2)	0.8 (0.2)	0.7 (0.2)

Abbreviations: ASM, appendicular skeletal muscle; BMI, body mass index; SD, standard deviation; SMI, skeletal muscular index.

Differences were observed in age distribution, body composition and muscle mass. ILAS participants were the oldest (mean age: 63.5 ± 8.5 years), whereas NILS‐LSA (56.8 ± 13.0 years) and NAHSIT (56.0 ± 17.4 years) included younger individuals, with NAHSIT recruiting 21.2% of participants under 40 years. Mean BMI was highest in NAHSIT (24.7 ± 4.1 kg/m^2^) and ILAS (24.6 ± 3.5 kg/m^2^) but lower in NILS‐LSA (22.8 ± 3.1 kg/m^2^).

In terms of muscle mass, ASM was highest in NAHSIT (18.1 ± 4.6 kg) and lowest in NILS‐LSA (17.3 ± 4.1 kg). SMI was comparable in ILAS (6.9 ± 1.1 kg/m^2^) and NAHSIT (6.9 ± 1.2 kg/m^2^) but lower in NILS‐LSA (6.7 ± 1.0 kg/m^2^).

### 3.2 Phase 1: External Validation of Existing NHANES‐Derived Equations Across Three Diverse Asian Cohorts (ILAS, NILS‐LSA and NAHSIT)

External validation performance varied across the three cohorts (Table 2). In ILAS, the *R* ^2^ values were moderate (*R* ^2^ = 0.52–0.80), with Equation 4 showing the lowest RMSE (1.88) and near‐zero median estimation errors. NILS‐LSA demonstrated higher *R* ^2^ values (0.62–0.88), with Equation 6 achieving the lowest RMSE (1.45) and minor estimation errors (−0.29 to 0.96) for Equations 4–6. NAHSIT showed the highest *R* ^2^ values (0.71–0.89), with Equation 4 performing best (RMSE = 1.52, median error: 0.45, IQR: 1.82). The comparison of actual SMI and estimated SMI by sex was presented in (Figures S2 and S3). The Bland–Altman plots indicated that Equations 4–6 demonstrated consistent performance across the three cohorts (Figure S4).

**TABLE 2:** Performance of the model in external validation across the three cohorts.

	ILAS			NILS‐LSA			NAHSIT^a^		
	*R* ^2^	RMSE (95% CI)	Median (IQR)^b^	*R* ^2^	RMSE (95% CI)	Median (IQR)^b^	*R* ^2^	RMSE (95% CI)	Median (IQR)^b^
Equation 3	0.52	2.89 (2.78–3.00)	2.32 (2.08)	0.62	2.54 (2.46–2.62)	2.20 (1.52)	0.71	2.41 (2.35–2.48)	1.87 (1.86)
Equation 4	0.80	1.88 (1.81–1.95)	0.89 (2.01)	0.86	1.51 (1.46–1.56)	0.96 (1.50)	0.89	1.52 (1.49–1.56)	0.45 (1.82)
Equation 5	0.77	1.98 (1.91–2.05)	0.93 (2.12)	0.86	1.54 (1.49–1.59)	0.93 (1.59)	0.87	1.62 (1.59–1.66)	0.48 (1.88)
Equation 6	0.77	1.99 (1.91–2.06)	−0.64 (2.18)	0.88	1.45 (1.40–1.49)	−0.29 (1.81)	0.81	1.99 (1.95–2.04)	−0.72 (2.22)

Abbreviations: IQR, interquartile range; RMSE, root mean square error.

^a^ We excluded implausible serum creatinine data in NAHSIT (*n* = 788), resulting in a final sample of 6065 participants for calculation using Equation 3.

^b^ The median and interquartile range of the differences between actual and estimated ASM.

For diagnostic performance using AWGS 2019 criteria (Table S2), ILAS showed moderate discriminative ability (AUC: 0.79–0.83 in males, 0.76–0.77 in females), with the highest values for Equations 3–5. NILS‐LSA demonstrated superior performance (AUC: 0.86–0.90 in males and 0.84–0.85 in females), with Equations 3–5 achieving AUC = 0.90 in males and Equations 3, 5 and 6 reaching AUC = 0.85 in females. NAHSIT exhibited high diagnostic performance (AUC: 0.84–0.91 in males and 0.88–0.89 in females), with Equation 4 showing the highest males AUC (0.91) and most equations achieving AUC = 0.89 in females.

### 3.3 Phase 2: Development of New Asian‐Specific Prediction Equations From NAHSIT

We developed equations using 6853 NAHSIT participants, with balanced characteristics between development and validation cohorts (Table S3). Following univariate regression (Table S4), multivariate regression (Table S5) and stepwise regression, we incorporated log age, sex, log height and log weight based on *R* ^2^ (0.90), AIC (−19 512) and BIC (−24 310) (Tables S6 and S7).

Among the developed equations (Table S8), Equation 4 demonstrated superior performance with *R* ^2^ = 0.90, RMSE = 1.45 and minimal prediction bias (median error: 0.06, IQR: 1.81). Equation 5 showed slightly inferior predictive performance (RMSE = 1.54, median error: 0.09, IQR: 1.79) (Table S9).

**FIGURE 1:**
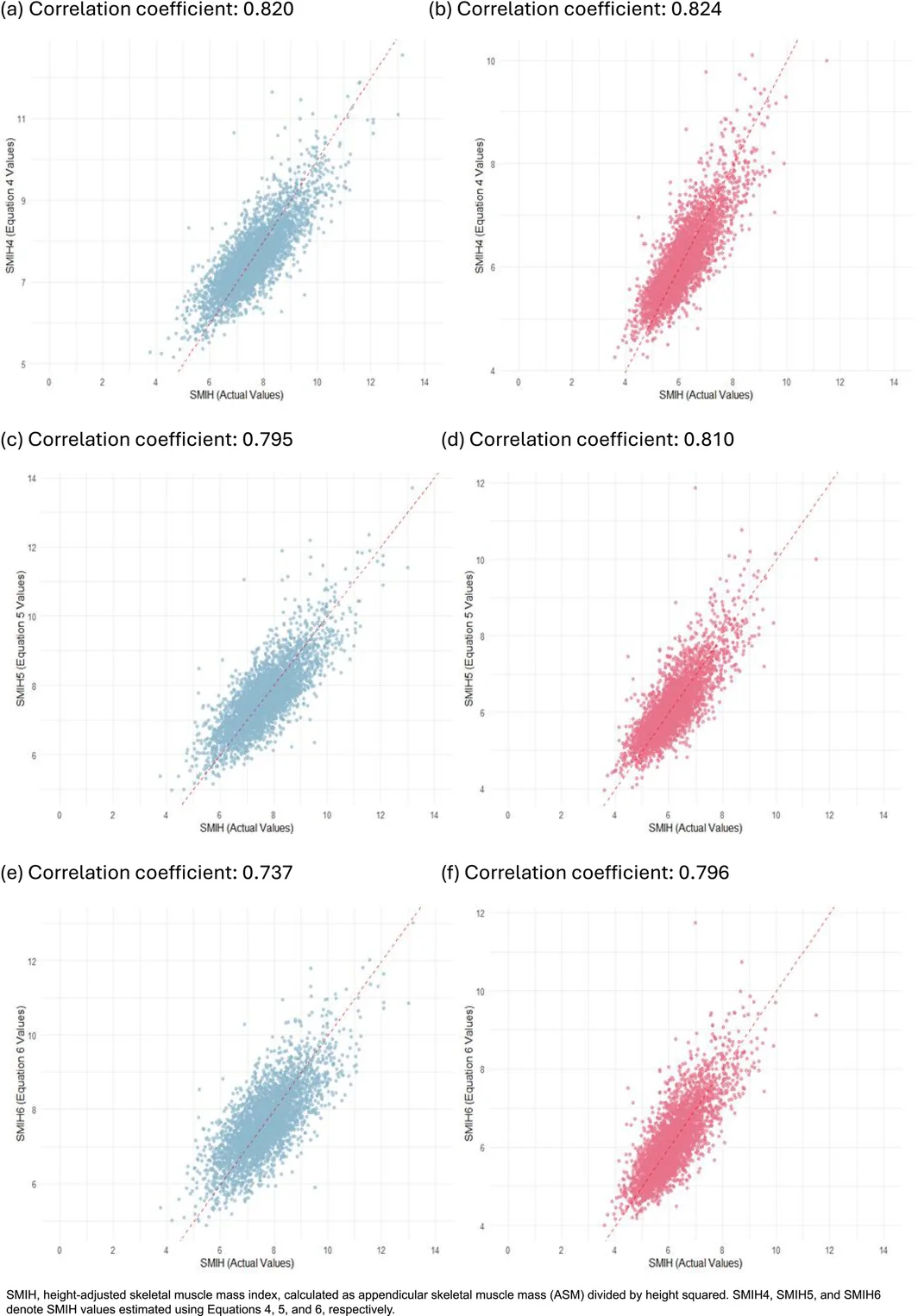
Correlation between the actual SMI and the estimated SMI in the NAHSIT cohort: Equation 4 in (a) male and (b) female; Equation 5 in (c) male and (d) female; and Equation 6 (e) male and (f) female.

Final equation parameters from the complete NAHSIT dataset (Table 3) showed high diagnostic performance. Equation 4 achieved the highest AUC (0.90 in both sexes), with sensitivity/specificity of 0.85/0.80 in males and 0.87/0.80 in females (Table 4). Scatter plots demonstrated strong correlations between estimated and actual SMI (*r* = 0.82 for both sexes) (Figure 1). The Bland–Altman plot demonstrated no systematic bias across equations, and no evident proportional bias was observed (Figures S5 and S6).

**TABLE 3:** Parameters of the newly developed equation and model fit derived from the complete dataset of the NAHSIT cohort.

Complete dataset	Formula	*R* ^2^	AIC	BIC
Equation 4	ASM = 0.01926437 * age^(−0.065719)^ * 0.832924731^Female^ * Weight^(0.698532)^ * Height^(0.841383)^	0.90	−27 846	−34 699
Equation 5	ASM = 1.259102292 * age^(−0.107785)^ * 0.791995951^Female^ * Weight^(0.767657)^	0.88	−27 024	−33 877
Equation 6	ASM = 0.725183176 * 0.798691912^Female^ * Weight^(0.796652)^	0.86	−25 684	−32 537

Abbreviations: AIC, Akaike information criterion; ASM, appendicular skeletal muscle; BIC, Bayesian information criterion.

**TABLE 4:** Diagnostic performance of newly developed equations for estimating ASM based on AWGS criteria in NAHSIT and NILS‐LSA cohort.

		NAHSIT								NILS‐LSA							
Estimated	Equation	Male				Female				Male				Female			
		AUC	Cutoff	Specificity	Sensitivity	AUC	Cutoff	Specificity	Sensitivity	AUC	Cutoff	Specificity	Sensitivity	AUC	Cutoff	Specificity	Sensitivity
AWGS SMI (kg/m^2^)	Equation 4	0.90	7.33	0.80	0.85	0.90	5.75	0.80	0.87	0.90	6.97	0.83	0.82	0.85	5.63	0.66	0.88
	Equation 5	0.89	7.19	0.84	0.77	0.90	5.73	0.79	0.86	0.90	6.94	0.83	0.82	0.85	5.42	0.81	0.73
	Equation 6	0.85	7.38	0.74	0.81	0.89	5.67	0.80	0.83	0.87	6.95	0.80	0.79	0.85	5.53	0.74	0.81

Abbreviations: ASM, appendicular skeletal muscle; AUC, area under curve; AWGS, Asian Working Group for Sarcopenia; SMI, skeletal muscular index.

### 3.4 Phase 3: External Validation Using the Final Equation in NILS‐LSA

External validation in the NILS‐LSA cohort (*n* = 3949) demonstrated robust cross‐population generalizability, with performance comparable to that of internal validation (Table 4). Equations 4 and 5 achieved the highest AUC values (0.90 in males and 0.85 in females). Correlations between estimated and actual SMI ranged from 0.75 to 0.84 across equations for both sexes, comparable to the NAHSIT cohort (Figure S7). The agreement assessed by the Bland–Altman plot showed minimal systematic bias across equations, with most of the observations falling within the range of 95% CI, and no evident proportional bias was observed (Figures S8 and S9).

## 4 Discussion

Our study represents the first comprehensive three‐phase investigation of muscle mass prediction equations in Asian populations, establishing validated models specifically calibrated for Asian body composition characteristics. Through systematic validation across 13 582 participants in Taiwan and Japan, we address a critical gap in sarcopenia screening such as limited DXA access and BIA instrumentation. Our findings demonstrate that Asian‐specific equations achieve superior performance and exhibit robust cross‐population generalizability. Based on a comprehensive evaluation across all three phases, we recommend Equation 4 as optimal for clinical practice, consistently demonstrating the strongest performance (Phase 1: *R* ^2^ = 0.80–0.89; Phase 2: *R* ^2^ = 0.90; and Phase 3: AUC = 0.90 in males and 0.85 in females). Critically, Equation 4 utilizes only readily accessible clinical parameters—age, sex, height and weight—without requiring serum creatinine measurements, enabling practical implementation in community settings without specialized equipment. Despite ongoing controversies, sarcopenia diagnosis remains predominantly contingent upon ASM quantification [26]; though both the EWGSOP and AWGS have proposed alternative paradigms (‘probable sarcopenia’ [15] and ‘possible sarcopenia’ [13], respectively) when precise ASM measurement is unfeasible. However, significant diagnostic challenges persist in Asia, where despite widespread awareness among healthcare professionals (97.2%), only 42.9% actually diagnosing sarcopenia in clinical practice [27], primarily due to limited access to DXA and BIA instrumentation. Alternative approaches have emerged, including CT‐based paraspinal muscle assessments [28], standardized questionnaires [29], simplified anthropometric assessment protocols [30] and machine learning models [31]. However, these methods often require specialized equipment, complex algorithms or additional biomarkers not routinely available in community settings, limiting their practical implementation for large‐scale sarcopenia screening where accessible diagnostic tools are most critically needed.

Complementing these advances, a prior systematic review identified numerous anthropometric equations for muscle mass prediction, finding excellent accuracy in healthy adults but limited validation in clinically relevant populations like older adults, those with obesity or malnourished individuals [32]. Although NHANES‐derived prediction models demonstrate strong accuracy with robust methodology, they significantly underrepresent Asian populations [17]. Alternative biomarker approaches such as the Japanese SONIC study's serum creatinine/cystatin C ratio showed limited correlation with skeletal muscle mass index and restricted clinical utility as cystatin C is seldom measured in routine practice [33]. Notably, Furushima et al. [34] previously developed anthropometric prediction equations for lean soft tissue mass in Japanese populations using DXA reference standards and readily available measurements (age, height, weight, waist circumference and handgrip strength), establishing important groundwork for population‐specific approaches in Asian cohorts.

Building upon this foundation, our study addresses these limitations and presents several key strengths that substantially advance sarcopenia assessment in Asian populations. This represents the first population‐based study to both validate existing equations and develop new prediction models specifically calibrated for Asian body composition characteristics, employing a multinational scope encompassing Taiwanese and Japanese populations and a population‐based design using nationally representative data. Our work encompasses the most extensive validation effort to date with 13 582 participants across three cohorts from Taiwan and Japan, including notable demographic diversity with 21.2% of participants under 40 years. The comprehensive three‐phase validation framework follows established clinical prediction model frameworks, demonstrating superior methodological rigor with readily available clinical data (using only routine creatinine measurements), DXA reference standards and superior diagnostic performance (*R* ^2^ = 0.90, AUC = 0.90) while maintaining practical clinical utility for community‐based screening where DXA access is limited. Rather than optimizing a single performance metric, this systematic three‐phase approach allowed us to assess how well existing equations perform across diverse Asian cohorts before developing population‐specific alternatives, identifying where existing equations hold, where they fall short and where population‐specific refinement adds value. Employing ethnicity‐specific calibration parameters, our prediction algorithm facilitates prospective assessments in community‐based contexts where DXA/BIA instrumentation remains inaccessible.

The key clinical utility of these equations lies in their ability to estimate ASM without the need for specialized imaging equipment, using only routinely collected parameters—age, sex, height and weight. This enables frontline healthcare providers, including general practitioners, nurses and community health workers, to screen for low muscle mass using measurements already collected during routine health assessments. Early identification enables timely referral, lifestyle interventions and nutritional support, all of which are critical for preventing functional decline in older adults. The equations' strong diagnostic performance (AUC = 0.90 for men and 0.85 for women) and validation across multiple Asian cohorts from Taiwan and Japan further support their reliability and generalizability for large‐scale sarcopenia screening across diverse Asian populations.

However, several limitations warrant consideration. Our study population primarily comprised community‐dwelling adults, potentially limiting generalizability to hospitalized populations. Additionally, the NILS‐LSA cohort used for external validation did not include participants under 40 years of age, which may limit the generalizability of our validation findings to younger adult populations. In addition, different DXA systems were used across cohorts. Whereas between‐device differences in lean mass estimation are well recognized, harmonizing equipment across independently established national cohorts is rarely feasible in cross‐national research. However, the impact of this limitation is likely minimal, as each cohort underwent independent model development and validation without data pooling, ensuring internal measurement consistency within each model. Furthermore, although DXA is clinically endorsed, MRI and CT provide superior muscle mass quantification by distinguishing intramuscular fat from contractile tissue [35]. Additionally, the cross‐sectional design prevents assessment of longitudinal muscle mass tracking. Despite these limitations, this study represents a significant milestone, delivering the first population‐based muscle mass prediction equations specifically developed and validated across multiple Asian populations. The robustly calibrated equations demonstrate strong diagnostic performance and practical utility for large‐scale sarcopenia screening in community settings with limited access to DXA. Future research should expand validation to additional Asian ethnicities, evaluate predictive value for adverse outcomes and assess performance in longitudinal monitoring and clinical populations.

## 5 Conclusions

Our three‐phase investigation has established and validated the first population‐based prediction equations for muscle mass estimation in Asian populations. The robust diagnostic performance and practicality of these equations make them valuable tools for sarcopenia screening, particularly in settings with limited access to DXA. These findings mark a significant advance in enabling early identification of sarcopenia and promoting healthy aging across Asian populations.

## Funding

This study was supported by the Taiwan National Science and Technology Council (NSTC 113‐2321‐B‐A49‐028‐ and NSTC 114‐2321‐B‐A49‐012‐) and the Interdisciplinary Research Center for Healthy Longevity of National Yang Ming Chiao Tung University.

## Conflicts of Interest

The authors declare no conflicts of interest.

## Supporting information


**Table S1:** Characteristics of the total population across the three cohorts, stratified by sex.
**Table S2:** Diagnostic performance of equations to estimate ASM based on AWGS criteria across three cohorts.
**Table S3:** Characteristics of total population of NAHSIT, stratified by training status.
**Table S4:** Univariate linear regression (development set).
**Table S5:** Multivariate linear regression (development set).
**Table S6:** Selection of statistical forms for key variables.
**Table S7:** Stepwise regression of log ASM in development set.
**Table S8:** Parameters of estimated equation and model fit from development set.
**Table S9:** Model performance of validation set from NAHSIT.
**Figure S1:** Study flowchart of newly developed equations from NAHSIT.
**Figure S2:** Comparison of actual SMI *in male* and the estimated SMI calculated by *Equation 3*: (a) ILAS (b) NILS‐LSA (c) NAHSIT; *Equation 4*: (d) ILAS (e) NILS‐LSA (f) NAHSIT; *Equation 5*: (g) ILAS (h) NILS‐LSA (i) NAHSIT; *Equation 6*: (j) ILAS (k) NILS‐LSA (l) NAHSIT.
**Figure S3:** Comparison of actual SMI *in female* and the estimated SMI calculated by *Equation 3*: (a) ILAS (b) NILS‐LSA (c) NAHSIT; *Equation 4*: (d) ILAS (e) NILS‐LSA (f) NAHSIT; *Equation 5*: (g) ILAS (h) NILS‐LSA (i) NAHSIT; *Equation 6*: (j) ILAS (k) NILS‐LSA (l) NAHSIT.
**Figure S4:** The Bland–Altman plot of ASM of existing NHANES‐derived equation: Equation 3 in (a) ILAS (b) NILS‐LSA (c) NAHSIT, Equation 4 in (d) ILAS (e) NILS‐LSA (f) NAHSIT, Equation 5 in (g) ILAS (h) NILS‐LSA (i) NAHSIT, Equation 6 in (j) ILAS (k) NILS‐LSA (l) NAHSIT.
**Figure S5:** The Bland–Altman plot of ASM of newly developed equation in NAHSIT cohort: Equation 4 in (a) male (b) female, Equation 5 in (c) male (d) female, Equation 6 (e) male (f) female.
**Figure S6:** The Bland–Altman plot of SMI of newly developed equation in NAHSIT cohort: Equation 4 in (a) male (b) female, Equation 5 in (c) male (d) female, Equation 6 (e) male (f) female.
**Figure S7:** Scatter plot between the actual SMI and the estimated SMI in NILS‐LSA cohort: Equation 4 in (a) male (b) female, Equation 5 in (c) male (d) female, Equation 6 (e) male (f) female.
**Figure S8:** The Bland–Altman plot of ASM of newly developed equation in NILS‐LSA cohort: Equation 4 in (a) male (b) female, Equation 5 in (c) male (d) female, Equation 6 (e) male (f) female.
**Figure S9:** The Bland–Altman plot of SMI of newly developed equation in NILS‐LSA cohort: Equation 4 in (a) male (b) female, Equation 5 in (c) male (d) female, Equation 6 (e) male (f) female.

## Data Availability

Data described in the manuscript, code book and analytic code will not be made available because only authorized investigators can access the data.
