# Supplementary material for: Population‐Based Prediction Equations for Appendicular Skeletal Muscle Mass: A Three‐Phase Validation Study in 13 582 Asian Adults
Source: J Cachexia Sarcopenia Muscle. 2026 Sep 6;17(5):e70360. doi: 10.1002/jcsm.70360 (PMC13547844; doi:10.1002/jcsm.70360)
Supplement: Supplementary file 1 [file JCSM-17-e70360-s001.docx]

**Table of Contents**

[**Supplementary information 1.** **Cohorts description**	3](#_Toc222848006)

[**Supplementary information 2.** **The formulation of the previous equations from Shi et al**	4](#_Toc222848007)

[**Supplementary Table 1. Characteristics of the total population across the three cohorts, stratified by sex**	5](#_Toc222848008)

[**Supplementary Table 2. Diagnostic performance of equations to estimate ASM based on AWGS criteria across three cohorts**	6](#_Toc222848009)

[**Supplementary Table 3. Characteristics of total population of NAHSIT, stratified by training status**	8](#_Toc222848010)

[**Supplementary Table 4. Univariate linear regression (development set)**	10](#_Toc222848011)

[**Supplementary Table 5. Multivariate linear regression (development set)**	12](#_Toc222848012)

[**Supplementary Table 6. Selection of statistical forms for key variables**	13](#_Toc222848013)

[**Supplementary Table 7.** **Stepwise regression of log ASM in development set**	14](#_Toc222848014)

[**Supplementary Table 8.** **Parameters of estimated equation and model fit from development set**	15](#_Toc222848015)

[**Supplementary Table 9. Model performance of validation set from NAHSIT**	16](#_Toc222848016)

[**Supplementary Figure 1. Study flowchart of newly developed equations from NAHSIT**	17](#_Toc222848017)

[17](#_Toc222848018)

[**Supplementary Figure 2. Comparison of actual SMI *in male* and the estimated SMI calculated by *Equation 3*: (a) ILAS (b) NILS-LSA (c) NAHSIT; *Equation 4*: (d) ILAS (e) NILS-LSA (f) NAHSIT; *Equation 5*: (g) ILAS (h) NILS-LSA (i) NAHSIT; *Equation 6*: (j) ILAS (k) NILS-LSA (l) NAHSIT**	18](#_Toc222848019)

[**Supplementary Figure 3. Comparison of actual SMI *in female* and the estimated SMI calculated by *Equation 3*: (a) ILAS (b) NILS-LSA (c) NAHSIT; *Equation 4*: (d) ILAS (e) NILS-LSA (f) NAHSIT; *Equation 5*: (g) ILAS (h) NILS-LSA (i) NAHSIT; *Equation 6*: (j) ILAS (k) NILS-LSA (l) NAHSIT**	22](#_Toc222848020)

[**Supplementary Figure 4. The Bland-Altman plot of ASM of existing NHANES-derived equation: Equation 3 in (a) ILAS (b) NILS-LSA (c) NAHSIT, Equation 4 in (d) ILAS (e) NILS-LSA (f) NAHSIT, Equation 5 in (g) ILAS (h) NILS-LSA (i) NAHSIT, Equation 6 in (j) ILAS (k) NILS-LSA (l) NAHSIT**	26](#_Toc222848021)

[26](#_Toc222848022)

[**Supplementary Figure 5. The Bland-Altman plot** **of ASM of newly developed equation in NAHSIT cohort: Equation 4 in (a) male (b) female, Equation 5 in (c) male (d) female, Equation 6 (e) male (f) female**	27](#_Toc222848023)

[**Supplementary Figure 6. The Bland-Altman plot** **of SMI of newly developed equation in NAHSIT cohort: Equation 4 in (a) male (b) female, Equation 5 in (c) male (d) female, Equation 6 (e) male (f) female**	28](#_Toc222848024)

[**Supplementary Figure 7. Scatter plot between the actual SMI and the estimated SMI in NILS-LSA cohort: Equation 4 in (a) male (b) female, Equation 5 in (c) male (d) female, Equation 6 (e) male (f) female**	29](#_Toc222848025)

[**Supplementary Figure 8. The Bland-Altman plot** **of ASM of newly developed equation in NILS-LSA cohort: Equation 4 in (a) male (b) female, Equation 5 in (c) male (d) female, Equation 6 (e) male (f) female**	31](#_Toc222848026)

[**Supplementary Figure 9. The Bland-Altman plot** **of SMI of newly developed equation in NILS-LSA cohort: Equation 4 in (a) male (b) female, Equation 5 in (c) male (d) female, Equation 6 (e) male (f) female**	32](#_Toc222848027)

**Supplementary information 1.** **Cohorts description**

I-Lan Longitudinal Aging Study (ILAS)

The I-Lan Longitudinal Aging Study (ILAS) is a population-based, prospective cohort study conducted in I-Lan County, Taiwan, which is designed to investigate the interrelationship between aging, sarcopenia, frailty, and cognitive decline. Participants were randomly selected from the household registration records of the county government in Yuanshan Township, I-Lan County. Eligible individuals were aged 50 years or older and were invited to participate via mail or telephone. Individuals with severe disability, dementia, communication difficulties, end-stage illness, or those residing in institutional care facilities were excluded from the study. Only those who were currently residing in Yuanshan Township with no immediate plans to move out were eligible for participation.

National Institute for Longevity Sciences-Longitudinal Study of Aging (NILS-LSA)

The National Institute for Longevity Sciences-Longitudinal Study of Aging (NILS-LSA) is a comprehensive Japanese population-based prospective cohort study that examines variables related to normal aging and age-related diseases. The study was initially launched with its first wave conducted between November 1997 and April 2000, recruiting 2,267 participants aged 40 to 79 years through age- and sex-stratified random sampling from Obu city and Higashiura town in Aichi Prefecture, Japan. Subsequent waves of data collection were systematically conducted: second wave (April 2000 - May 2002), third wave (May 2002 - May 2004), fourth wave (June 2004 - July 2006), fifth wave (July 2006 - July 2008), sixth wave (July 2008 - July 2010), and seventh wave (July 2010 - July 2012). The research area was specifically chosen for its representation of typical Japanese lifestyle, featuring both urban and rural characteristics. The study design ensures equal representation across age decades (40s, 50s, 60s, 70s) and sex, with participants undergoing comprehensive biennial examinations that include over 1,000 different measurements spanning clinical evaluations, anthropometry, body composition, physical functions, physical activities, psychological assessments, nutritional analysis, and molecular epidemiology. To maintain the cohort's age-sex distribution, when participants aged ≤79 years could not attend follow-up sessions, they were replaced by randomly recruited new participants matched for age and sex, while 40-year-olds were recruited annually.

The Nutrition and Health Survey in Taiwan (NAHSIT)

The Nutrition and Health Survey in Taiwan (NAHSIT) is a nationwide, population-based survey originally managed by the Bureau of Food and Drug, Department of Health and later transferred to the Health Promotion Administration (HPA) in 2013. A major redesign was introduced to establish a continuous monitoring system for tracking the health and nutritional status of Taiwanese citizens, providing essential data for public health policymaking. NAHSIT utilizes a multistage, stratified, clustered probability sampling design, ensuring a representative sample of Taiwan’s civilian, non-institutionalized population. Data collection includes face-to-face interviews conducted at participants' homes, while health measurements take place at designated local survey sites or through mobile health stations traveling across Taiwan. NAHSIT has conducted three recent waves: 2013–2016, 2017–2020, and 2021–2024 (ongoing). NAHSIT examines lifestyle behaviors, health conditions, and disease prevalence, focusing on behavioral indicators such as dietary habits, physical activity, weight management, and substance use (tobacco, alcohol, betel nuts), as well as health outcomes, including obesity, body composition, metabolic syndrome, cardiovascular diseases, diabetes, kidney disease, respiratory conditions, and environmental exposure.

**Supplementary information 2.** **The formulation of the previous equations from Shi et al**

|  | Formula |
| --- | --- |
| Equation 3 | ASM=0.578×0.997^age^×0.831^[female]^×1.005^height^×weight^0.674^×Cr^0.129^ |
| Equation 4 | ASM=0.485×0.998^age^×0.814^[female]^×1.006^height^×weight^0.680^ |
| Equation 5 | ASM=0.939×0.997^age^×0.764^[female]^×weight^0.774^ |
| Equation 6 | ASM=1.005×0.753^[female]^×weight^0.736^ |

**Supplementary Table 1. Characteristics of the total population across the three cohorts, stratified by sex**

| Variables | | Male | | | Female | | |
| --- | --- | --- | --- | --- | --- | --- | --- |
|  |  | ILAS (N=1,355) | NILS-LSA (N=1,967) | NAHSIT (N=3,399) | ILAS (N=1,425) | NILS-LSA (N=1,982) | NAHSIT (N=3,454) |
| Age (years), mean (SD) | | 64.2 (8.8) | 56.4 (12.9) | 56.9 (17.6) | 62.8 (8.0) | 57.2 (13.1) | 55.1 (17.1) |
|  | <25, n (%) | 0 (0.0) | 0 (0.0) | 173 (5.1) | 0 (0.0) | 0 (0.0) | 186 (5.5) |
|  | 25-29, n (%) | 0 (0.0) | 0 (0.0) | 192 (5.6) | 0 (0.0) | 0 (0.0) | 221 (6.5) |
|  | 30-34, n (%) | 0 (0.0) | 0 (0.0) | 165 (4.9) | 0 (0.0) | 0 (0.0) | 155 (4.6) |
|  | 35-39, n (%) | 0 (0.0) | 0 (0.0) | 165 (4.9) | 0 (0.0) | 0 (0.0) | 197 (5.7) |
|  | 40-44, n (%) | 0 (0.0) | 532 (27.0) | 178 (5.2) | 0 (0.0) | 508 (25.6) | 204 (5.9) |
|  | 45-49, n (%) | 4 (0.30) | 206 (10.5) | 181 (5.3) | 9 (0.6) | 222 (11.2) | 252 (7.4) |
|  | 50-54, n (%) | 202 (14.9) | 202 (10.3) | 240 (7.1) | 243 (17.1) | 178 (9.0) | 278 (8.2) |
|  | 55-59, n (%) | 264 (19.5) | 213 (10.8) | 321 (9.4) | 307 (21.5) | 158 (8.0) | 318 (9.4) |
|  | 60-64, n (%) | 277 (20.4) | 175 (8.9) | 332 (9.8) | 289 (20.3) | 208 (10.5) | 321 (9.4) |
|  | 65-69, n (%) | 226 (16.7) | 156 (7.9) | 583 (17.2) | 259 (18.2) | 163 (8.2) | 572 (16.8) |
|  | 70-74, n (%) | 195 (14.4) | 297 (15.1) | 364 (10.7) | 199 (14.0) | 318 (16.0) | 367 (10.8) |
|  | 75-79, n (%) | 114 (8.4) | 180 (9.2) | 266 (7.8) | 83 (5.8) | 222 (11.2) | 234 (6.9) |
|  | 80+, n (%) | 73 (5.4) | 6 (0.3) | 239 (7.0) | 36 (2.5) | 5 (0.3) | 149 (4.4) |
| Height (cm), mean (SD) | | 165.2 (6.0) | 165.8 (6.8) | 166.9 (7.0) | 154.0 (5.6) | 152.3 (6.4) | 155.2 (6.5) |
| Weight (kg), mean (SD) | | 68.0 (10.2) | 63.4 (9.7) | 69.8 (12.4) | 57.7 (9.2) | 52.4 (8.2) | 58.7 (10.9) |
| BMI （kg/m^2^）, mean (SD) | | 24.9 (3.2) | 23.0 (2.9) | 25.0 (3.8) | 24.3 (3.6) | 22.6 (3.3) | 24.4 (4.4) |
|  | ≤18.4, n (%) | 12 (0.9) | 98 (5.0) | 88 (2.6) | 32 (2.2) | 143 (7.2) | 154 (4.5) |
|  | 18.5-23.9, n (%) | 550 (40.6) | 1196 (60.8) | 1279 (37.6) | 704 (49.4) | 1275 (64.3) | 1593 (46.1) |
|  | 24.0-29.9, n (%) | 714 (52.7) | 650 (33.0) | 1655 (48.7) | 590 (41.4) | 505 (25.5) | 1279 (37.0) |
|  | 30.0-39.9, n (%) | 78 (5.8) | 22 (1.1) | 307 (9.0) | 98 (6.9) | 57 (2.9) | 342 (9.9) |
|  | ≥40, n (%) | 1 (0.1) | 1 (0.1) | 70 (2.1) | 1 (0.1) | 2 (0.1) | 86 (2.5) |
| ASM (kg), mean (SD) | | 21.3 (2.8) | 20.4 (3.0) | 21.4 (3.8) | 14.5 (2.0) | 14.1 (2.0) | 14.9 (2.4) |
| SMI (kg/m^2^), mean (SD) | | 7.8 (0.8) | 7.4 (0.8) | 7.7 (1.1) | 6.1 (0.70) | 6.1 (0.7) | 6.2 (0.9) |
| ASM/BMI, [kg/(kg/m^2^)], mean (SD) | | 0.9 (0.1) | 0.9 (0.1) | 0.9 (0.1) | 0.6 (0.1) | 0.6 (0.1) | 0.6 (0.1) |

**Supplementary Table 2. Diagnostic performance of equations to estimate ASM based on AWGS criteria across three cohorts**

| Equation | Male | | | | Female | | | |
| --- | --- | --- | --- | --- | --- | --- | --- | --- |
|  | AUC | Cutoff | Specificity | Sensitivity | AUC | Cutoff | Specificity | Sensitivity |
| ILAS | | | | | | | | |
| Equation 3 | 0.83 | 6.53 | 0.72 | 0.81 | 0.77 | 5.09 | 0.67 | 0.78 |
| Equation 4 | 0.83 | 7.02 | 0.77 | 0.73 | 0.77 | 5.64 | 0.63 | 0.80 |
| Equation 5 | 0.83 | 7.10 | 0.73 | 0.79 | 0.77 | 5.53 | 0.65 | 0.78 |
| Equation 6 | 0.79 | 7.95 | 0.69 | 0.77 | 0.76 | 6.14 | 0.60 | 0.81 |
| NILS-LSA | | | | | | | | |
| Equation 3 | 0.90 | 6.35 | 0.84 | 0.80 | 0.85 | 4.92 | 0.72 | 0.81 |
| Equation 4 | 0.90 | 6.91 | 0.83 | 0.81 | 0.84 | 5.36 | 0.72 | 0.81 |
| Equation 5 | 0.90 | 6.90 | 0.83 | 0.83 | 0.85 | 5.32 | 0.73 | 0.80 |
| Equation 6 | 0.86 | 7.49 | 0.81 | 0.76 | 0.85 | 5.74 | 0.72 | 0.81 |
| NAHSIT | | | | | | | | |
| Equation 3 | 0.88 | 6.61 | 0.80 | 0.81 | 0.89 | 5.01 | 0.81 | 0.81 |
| Equation 4 | 0.91 | 7.32 | 0.76 | 0.89 | 0.89 | 5.60 | 0.76 | 0.89 |
| Equation 5 | 0.90 | 7.23 | 0.80 | 0.83 | 0.89 | 5.46 | 0.79 | 0.84 |
| Equation 6 | 0.84 | 7.95 | 0.73 | 0.80 | 0.88 | 5.87 | 0.77 | 0.84 |

ASM= appendicular skeletal muscle; SMI=skeletal muscular index; AWGS= Asian Working Group for Sarcopenia; AUC=area under curve

Equation 3: ASM = 0.578 × 0.997^age^ × 0.831^[female]^ × 1.005^height^ × weight0.674 × Cr^0.129^; Equation 4: ASM = 0.485 × 0.998^age^ × 0.814^[female]^ × 1.006^height^ × weight^0.680^; Equation 5: ASM = 0.939 × 0.997^age^ × 0.764^[female]^ × weight^0.774^; Equation 6: ASM = 1.005 × 0.753^[female]^ × weight^0.736^

**Supplementary Table 3. Characteristics of total population of NAHSIT, stratified by training status**

| Variables | | Overall N=6,853 | Development N=4,798 | Validation N=2,055 | P-Value |
| --- | --- | --- | --- | --- | --- |
| Age (years), mean (SD) | | 55.96 (17.35) | 56.16 (17.30) | 55.49 (17.46) | 0.425 |
|  | <25, n (%) | 359 (5.24%) | 249 (5.19%) | 110 (5.35%) | 0.760 |
|  | 25-29, n (%) | 413 (6.03%) | 283 (5.90%) | 130 (6.33%) |  |
|  | 30-34, n (%) | 320 (4.67%) | 214 (4.46%) | 106 (5.16%) |  |
|  | 35-39, n (%) | 362 (5.28%) | 249 (5.19%) | 113 (5.50%) |  |
|  | 40-44, n (%) | 382 (5.57%) | 276 (5.75%) | 106 (5.16%) |  |
|  | 45-49, n (%) | 433 (6.32%) | 293 (6.11%) | 140 (6.81%) |  |
|  | 50-54, n (%) | 518 (7.56%) | 354 (7.38%) | 164 (7.98%) |  |
|  | 55-59, n (%) | 639 (9.32%) | 454 (9.46%) | 185 (9.00%) |  |
|  | 60-64, n (%) | 653 (9.53%) | 471 (9.82%) | 182 (8.86%) |  |
|  | 65-69, n (%) | 1155 (16.85%) | 809 (16.86%) | 346 (16.84%) |  |
|  | 70-74, n (%) | 731 (10.67%) | 508 (10.59%) | 223 (10.85%) |  |
|  | 75-79, n (%) | 500 (7.30%) | 361 (7.52%) | 139 (6.76%) |  |
|  | 80+, n (%) | 388 (5.66%) | 277 (5.77%) | 111 (5.40%) |  |
| Female, n (%) | | 3454 (50.40%) | 2438 (50.81%) | 1016 (49.44%) | 0.298 |
| Height (cm), mean (SD) | | 161.00 (8.91) | 160.92 (8.93) | 161.18 (8.87) | 0.451 |
| Weight (kg), mean (SD) | | 64.23 (12.94) | 64.15 (12.86) | 64.43 (13.13) | 0.869 |
| BMI （kg/m^2^）, mean (SD) | | 24.71 (4.11) | 24.70 (4.08) | 24.73 (4.20) | 0.878 |
|  | ≤18.4, n (%) | 242 (3.53%) | 172 (3.58%) | 70 (3.41%) | 0.675 |
|  | 18.5-23.9, n (%) | 2872 (41.91%) | 2022 (42.14%) | 850 (41.36%) |  |
|  | 24.0-29.9, n (%) | 2934 (42.81%) | 2056 (42.85%) | 878 (42.73%) |  |
|  | 30.0-39.9, n (%) | 649 (9.47%) | 438 (9.13%) | 211 (10.27%) |  |
|  | ≥40.0, n (%) | 156 (2.28%) | 110 (2.29%) | 46 (2.24%) |  |
| ASM (kg), mean (SD) | | 18.12 (4.57) | 18.07 (4.55) | 18.22 (4.61) | 0.702 |
| SMI (kg/m^2^), mean (SD) | | 6.90 (1.22) | 6.89 (1.22) | 6.93 (1.24) | 0.448 |
| ASM/BMI, [kg/(kg/m^2^)], mean (SD) | | 0.74 (0.16) | 0.74 (0.16) | 0.74 (0.17) | 0.410 |
| Serum creatinine (mg/dL), mean (SD) | | 0.72 (0.42) | 0.72 (0.44) | 0.72 (0.38) | 0.791 |
| Serum phosphorus (mg/dL), mean (SD) | | 3.60 (0.49) | 3.61 (0.49) | 3.58 (0.49) | 0.465 |
| Serum calcium(mg/dL), mean (SD) | | 9.33 (0.42) | 9.32 (0.41) | 9.35 (0.44) | 0.151 |
| Serum BUN (mg/dL), mean (SD) | | 14.93 (5.79) | 14.96 (5.88) | 14.87 (5.59) | 0.650 |
| Urine protein | |  |  |  | 0.306 |
| Negative | | 2594 (37.85%) | 1851 (38.58%) | 743 (36.16%) |  |
| 1+ | | 4028 (58.78%) | 2782 (57.98%) | 1246 (60.63%) |  |
| 2+ | | 132 (1.93%) | 92 (1.92%) | 40 (1.95%) |  |
| 3+ | | 72 (1.05%) | 50 (1.04%) | 22 (1.07%) |  |
| ≥4+ | | 27 (0.39%) | 23 (0.48%) | 4 (0.19%) |  |
| Urine creatinine (mg/dL) | | 95.60 (62.71) | 95.05 (62.34) | 96.89 (63.55) | 0.528 |

*SD=standard deviation; BMI=body mass index; ASM= appendicular skeletal muscle; SMI=skeletal muscular index; BUN= blood urea nitrogen

* The missing number of serum creatinine was 62 (2.17%); serum phosphorus was 1800 (26.27%); serum BUN was 62 (2.17%); Urine creatinine was 228 (3.33%)

**Supplementary Table 4. Univariate linear regression (development set)**

| Development set | Scale | ASM (Natural scale) | | |  | | | ASM (Log scale) | | |  |
| --- | --- | --- | --- | --- | --- | --- | --- | --- | --- | --- | --- |
|  |  | Estimate | SE | P-value | |  | Estimate | | SE | P-value | |
| Age (yrs) | Natural scale | -0.059 | 0.004 | <.0001 | |  | -0.003 | | 0.000 | <.0001 | |
|  | Log scale | -2.589 | 0.176 | <.0001 | |  | -0.128 | | 0.010 | <.0001 | |
| Female (1) | Natural scale | -6.574 | 0.091 | <.0001 | |  | -0.364 | | 0.005 | <.0001 | |
| Height (cm) | Natural scale | 0.394 | 0.005 | <.0001 | |  | 0.022 | | 0.000 | <.0001 | |
|  | Log scale | 63.013 | 0.756 | <.0001 | |  | 3.46 | | 0.040 | <.0001 | |
| Weight (kg) | Natural scale | 0.294 | 0.003 | <.0001 | |  | 0.016 | | 0.000 | <.0001 | |
|  | Log scale | 19.271 | 0.189 | <.0001 | |  | 1.055 | | 0.010 | <.0001 | |
| Serum creatinine  (mg/dL) | Natural scale | 0.579 | 0.152 | 0.0001 | |  | 0.036 | | 0.008 | <.0001 | |
|  | Log scale | -0.518 | 0.049 | <.0001 | |  | -0.027 | | 0.003 | <.0001 | |
| Serum urea nitrogen (mg/dL) | Natural scale | 0.011 | 0.011 | 0.315 | |  | 0.000 | | 0.000 | 0.191 | |
|  | Log scale | 0.570 | 0.202 | 0.005 | |  | 0.036 | | 0.011 | 0.001 | |
| Serum calcium  (mg/dL) | Natural scale | 0.903 | 0.162 | <.0001 | |  | 0.045 | | 0.009 | <.0001 | |
|  | Log scale | 8.581 | 1.524 | <.0001 | |  | 0.428 | | 0.082 | <.0001 | |
| Serum phosphorus  (mg/dL) | Natural scale | -1.838 | 0.167 | <.0001 | |  | -0.107 | | 0.009 | <.0001 | |
|  | Log scale | -6.557 | 0.590 | <.0001 | |  | -0.38 | | 0.03 | <.0001 | |
| Urine protein (1) | Natural scale | 0.227 | 0.193 | 0.240 | |  | 0.014 | | 0.011 | 0.171 | |
| Urine creatinine (mg/dL) | Natural scale | 0.023 | 0.001 | <.0001 | |  | 0.001 | | 0.000 | <.0001 | |
|  | Log scale | 2.331 | 0.099 | <.0001 | |  | 0.126 | | 0.005 | <.0001 | |

*SD=standard deviation; SE=standard error; BMI=body mass index; ASM= appendicular skeletal muscle; SMI=skeletal muscular index; BUN= blood urea nitrogen

Variables with p-value <0.01 for at least 1 model were selected into the multiple linear regression

| Development set | Scale | ASM (Natural scale) | | | | | |  | ASM (Log scale) | | | | | |
| --- | --- | --- | --- | --- | --- | --- | --- | --- | --- | --- | --- | --- | --- | --- |
|  |  | Estimate | SE | P-value | Estimate | SE | P-value |  | Estimate | SE | P-value | Estimate | SE | P-value |
| Age (yrs) | Natural scale | -0.0267 | 0.002 | <.0001 |  |  |  |  | -0.001 | 0.000 | <.0001 |  |  |  |
|  | Log scale |  |  |  | -1.371 | 0.114 | <.0001 |  |  |  |  | -0.059 | 0.006 | <.0001 |
| Female (1) | Natural scale | -3.048 | 0.085 | <.0001 | -2.968 | 0.087 | <.0001 |  | -0.170 | 0.005 | <.0001 | -0.165 | 0.004 | <.0001 |
| Height (cm) | Natural scale | 0.093 | 0.005 | <.0001 |  |  |  |  | 0.005 | 0.000 | <.0001 |  |  |  |
|  | Log scale |  |  |  | 14.436 | 0.854 | <.0001 |  |  |  |  | 0.792 | 0.042 | <.0001 |
| Weight (kg) | Natural scale | 0.203 | 0.003 | <.0001 |  |  |  |  | 0.011 | 0.000 | <.0001 |  |  |  |
|  | Log scale |  |  |  | 13.540 | 0.181 | <.0001 |  |  |  |  | 0.733 | 0.009 | <.0001 |
| Serum creatinine  (mg/dL) | Natural scale | -0.193 | 0.067 | 0.004 |  |  |  |  | -0.007 | 0.004 | 0.068 |  |  |  |
|  | Log scale |  |  |  | -0.089 | 0.021 | <.0001 |  |  |  |  | -0.004 | 0.001 | 0.0005 |
| Serum urea nitrogen  (mg/dL) | Natural scale | 0.007 | 0.006 | 0.233 |  |  |  |  | 0.000 | 0.000 | 0.500 |  |  |  |
|  | Log scale |  |  |  | 0.061 | 0.102 | 0.549 |  |  |  |  | 0.001 | 0.005 | 0.815 |
| Serum calcium  (mg/dL) | Natural scale | 0.059 | 0.080 | 0.458 |  |  |  |  | 0.000 | 0.004 | 0.953 |  |  |  |
|  | Log scale |  |  |  | 0.341 | 0.767 | 0.657 |  |  |  |  | -0.008 | 0.038 | 0.838 |
| Serum phosphorus  (mg/dL) | Natural scale | 0.047 | 0.063 | 0.459 |  |  |  |  | -0.002 | 0.003 | 0.613 |  |  |  |
|  | Log scale |  |  |  | 0.447 | 0.232 | 0.055 |  |  |  |  | 0.011 | 0.011 | 0.341 |
| Urine creatinine  (mg/dL) | Natural scale | 0.000 | 0.000 | 0.123 |  |  |  |  | 0.000 | 0.000 | 0.092 |  |  |  |
|  | Log scale |  |  |  | 0.087 | 0.053 | 0.099 |  |  |  |  | 0.006 | 0.003 | 0.019 |

**Supplementary Table 5. Multivariate linear regression (development set)**

*SD=standard deviation; SE=standard error; BMI=body mass index; ASM= appendicular skeletal muscle; SMI=skeletal muscular index; BUN= blood urea nitrogen

Variables with p-value <0.001 for all four models were included in the stepwise regression

**Supplementary Table 6. Selection of statistical forms for key variables**

| Development set | ASM (Natural scale) | | | |  | ASM (Log scale) | | |  |
| --- | --- | --- | --- | --- | --- | --- | --- | --- | --- |
|  | R^2^ | AIC | BIC |  | R^2^ | | AIC | BIC | |
| All in natural scale | 0.89 | 8802.14 | 4004.15 |  | 0.89 | | -19249 | -24047 | |
| Age transformed to log scale | 0.89 | 8858.39 | 4060.40 |  | 0.89 | | -19193 | -23991 | |
| Height transformed to log scale | 0.89 | 8840.54 | 4042.55 |  | 0.89 | | -19250 | -24048 | |
| Weight transformed to log scale | 0.88 | 9130.31 | 4332.32 |  | 0.90 | | -19579 | -24377 | |
| Age, height transformed to log scale | 0.89 | 8897.60 | 4099.61 |  | 0.89 | | -19195 | -23993 | |
| Height, weight transformed to log scale | 0.88 | 9175.55 | 4377.57 |  | 0.90 | | -19569 | -24367 | |
| Age, weight transformed to log scale | 0.88 | 9219.86 | 4421.87 |  | 0.90 | | -19522 | -24320 | |
| Age, height, weight transformed to log scale | 0.88 | 9226.56 | 4428.57 |  | 0.90 | | -19512 | -24310 | |

ASM= appendicular skeletal muscle; AIC= Akaike information criterion; BIC= Bayesian information criterion

**Supplementary Table 7.** **Stepwise regression of log ASM in development set**

| Development set | Scale | Estimate | SE | P-value |
| --- | --- | --- | --- | --- |
| Intercept |  | -4.064068 | 0.171132 |  |
| Age | log scale | -0.061773 | 0.003589 | <.0001 |
| Sex | natural scale | -0.180723 | 0.003289 | <.0001 |
| Height | log scale | 0.863920 | 0.033858 | <.0001 |
| Weight | log scale | 0.694509 | 0.007209 | <.0001 |

ASM= appendicular skeletal muscle; SE=standard error

**Supplementary Table 8.** **Parameters of estimated equation and model fit from development set**

| Development set | Formula | R^2^ | AIC | BIC |
| --- | --- | --- | --- | --- |
| Equation 4 | ASM= 0.017178993 *age (-0.061773)* 0.833465474 ^Female^ *Weight(0.694509) *Height(0.863920) | 0.90 | -19512 | -24310 |
| Equation 5 | ASM= 1.250829586 *age (-0.104845)* 0.792462574^Female^ *Weight(0.766364) | 0.88 | -18902 | -23700 |
| Equation 6 | ASM=0.725941388* 0.799792468 ^Female^ *Weight (0.796123) | 0.86 | -18025 | -22823 |

ASM= appendicular skeletal muscle; AIC= Akaike information criterion; BIC= Bayesian information criterion

**Supplementary Table 9. Model performance of validation set from NAHSIT**

| Validation set | NAHSIT | | |
| --- | --- | --- | --- |
|  | R^2^ | RMSE (95% CI) | Median (IQR)^a^ |
| Equation 4 | 0.90 | 1.45 (1.39-1.52) | 0.06 (1.81) |
| Equation 5 | 0.89 | 1.54 (1.47-1.61) | 0.09 (1.79) |
| Equation 6 | 0.86 | 1.74 (1.67-1.82) | 0.11 (2.04) |

NAHSIT= Nutrition and Health Survey in Taiwan; RMSE= root mean square error; IQR=Interquartile range

^a^ The median and interquartile range (IQR) of the differences between actual and estimated ASM

**Supplementary Figure 1. Study flowchart of newly developed equations from NAHSIT**

NAHSIT study across the 2013–2016 and 2017–2020 waves

(N = 23,192)

Eligible participants

N=6,853

Aged ≤18, n=10,431

Did not participate in physical examination, n=770

Missing BMI, BH, BW, or DXA assessment, n=5,138

Development set (70%)

N=4,798

Validation set (30%)

N=2,055

**Supplementary Figure 2.** **Comparison of actual SMI *in male* and the estimated SMI calculated by *Equation 3*: (a) ILAS (b) NILS-LSA (c) NAHSIT; *Equation 4*: (d) ILAS (e) NILS-LSA (f) NAHSIT; *Equation 5*: (g) ILAS (h) NILS-LSA (i) NAHSIT; *Equation 6*: (j) ILAS (k) NILS-LSA (l) NAHSIT**

(a) Correlation coefficient: 0.6073

(b) Correlation coefficient: 0.8303

(c) Correlation coefficient: 0.7445

(d) Correlation coefficient: 0.6079

(e) Correlation coefficient: 0.8341

(f) Correlation coefficient: 0.8263

(g) Correlation coefficient: 0.6084

(h) Correlation coefficient: 0.8322

(i) Correlation coefficient: 0.8080

(j) Correlation coefficient: 0.5531

(k) Correlation coefficient: 0.7617

(l) Correlation coefficient: 0.7188

SMI=skeletal muscle index; ILAS= I-Lan Longitudinal Aging Study; NILS-LSA= National Institute for Longevity Sciences-Longitudinal Study of Aging; NAHSIT= Nutrition and Health Survey in Taiwan

SMIH, height-adjusted skeletal muscle mass index, calculated as appendicular skeletal muscle mass (ASM) divided by height squared. SMIH4, SMIH5, and SMIH6 denote SMIH values estimated using Equations 4, 5, and 6, respectively.

**Supplementary Figure 3. Comparison of actual SMI *in female* and the estimated SMI calculated by *Equation 3*: (a) ILAS (b) NILS-LSA (c) NAHSIT; *Equation 4*: (d) ILAS (e) NILS-LSA (f) NAHSIT; *Equation 5*: (g) ILAS (h) NILS-LSA (i) NAHSIT; *Equation 6*: (j) ILAS (k) NILS-LSA (l) NAHSIT**

(a) Correlation coefficient: 0.591

(b) Correlation coefficient: 0.7645

(c) Correlation coefficient: 0.7627

(d) Correlation coefficient: 0.5912

(e) Correlation coefficient: 0.7726

(f) Correlation coefficient: 0.8148

(g) Correlation coefficient: 0.5808

(h) Correlation coefficient: 0.7717

(i) Correlation coefficient: 0.8076

(j) Correlation coefficient: 0.5724

(k) Correlation coefficient: 0.7717

(l) Correlation coefficient: 0.7856

SMI=skeletal muscle index; ILAS= I-Lan Longitudinal Aging Study; NILS-LSA= National Institute for Longevity Sciences-Longitudinal Study of Aging; NAHSIT= Nutrition and Health Survey in Taiwan

SMIH, height-adjusted skeletal muscle mass index, calculated as appendicular skeletal muscle mass (ASM) divided by height squared. SMIH4, SMIH5, and SMIH6 denote SMIH values estimated using Equations 4, 5, and 6, respectively.

**Supplementary Figure 4. The Bland-Altman plot of ASM of existing NHANES-derived equation: Equation 3 in (a) ILAS (b) NILS-LSA (c) NAHSIT, Equation 4 in (d) ILAS (e) NILS-LSA (f) NAHSIT, Equation 5 in (g) ILAS (h) NILS-LSA (i) NAHSIT,****Equation 6 in (j) ILAS (k) NILS-LSA (l) NAHSIT**

SMI=skeletal muscle index; ILAS= I-Lan Longitudinal Aging Study; NILS-LSA= National Institute for Longevity Sciences-Longitudinal Study of Aging; NAHSIT= Nutrition and Health Survey in Taiwan

**Supplementary Figure 5. The Bland-Altman plot** **of ASM of newly developed equation in NAHSIT cohort: Equation 4 in (a) male (b) female, Equation 5 in (c) male (d) female, Equation 6 (e) male (f) female**

ASM= appendicular skeletal muscle mass; NAHSIT= Nutrition and Health Survey in Taiwan

**Supplementary Figure 6. The Bland-Altman plot** **of SMI of newly developed equation in NAHSIT cohort: Equation 4 in (a) male (b) female, Equation 5 in (c) male (d) female, Equation 6 (e) male (f) female**

SMI=skeletal muscle index; NAHSIT= Nutrition and Health Survey in Taiwan

**Supplementary Figure 7. Scatter plot between the actual SMI and the estimated SMI in NILS-LSA cohort: Equation 4 in (a) male (b) female, Equation 5 in (c) male (d) female, Equation 6 (e) male (f) female**

(a) Correlation coefficient: 0.8389

(b) Correlation coefficient: 0.7834

(c) Correlation coefficient: 0.824

(d) Correlation coefficient: 0.7734

(e) Correlation coefficient: 0.7806

(f) Correlation coefficient: 0.7558

SMI=skeletal muscle index; NILS-LSA= National Institute for Longevity Sciences-Longitudinal Study of Aging

SMIH, height-adjusted skeletal muscle mass index, calculated as appendicular skeletal muscle mass (ASM) divided by height squared. SMIH4, SMIH5, and SMIH6 denote SMIH values estimated using Equations 4, 5, and 6, respectively.

**Supplementary Figure 8. The Bland-Altman plot** **of ASM of newly developed equation in NILS-LSA cohort: Equation 4 in (a) male (b) female, Equation 5 in (c) male (d) female, Equation 6 (e) male (f) female**

ASM= appendicular skeletal muscle mass; NILS-LSA= National Institute for Longevity Sciences-Longitudinal Study of Aging

**Supplementary Figure 9. The Bland-Altman plot** **of SMI of newly developed equation in NILS-LSA cohort: Equation 4 in (a) male (b) female, Equation 5 in (c) male (d) female, Equation 6 (e) male (f) female**

SMI=skeletal muscle index; NILS-LSA= National Institute for Longevity Sciences-Longitudinal Study of Aging
